# Genome-Wide Identification, Characterization, and Expression Analysis of CHS Gene Family Members in *Chrysanthemum nankingense*

**DOI:** 10.3390/genes13112145

**Published:** 2022-11-18

**Authors:** Lili Zhu, Yuqing Ding, Shunxiang Wang, Zhimin Wang, Liping Dai

**Affiliations:** 1 Academy of Chinese Medical Sciences, Henan University of Chinese Medicine, Zhengzhou 450046, China; 2 Engineering Center for Comprehensive Development and Utilization of Authentic Medicinal Materials in Henan Province, Zhengzhou 450046, China; 3 Institute of Chinese Materia Medica, China Academy of Chinese Medical Sciences, Beijing 100700, China

**Keywords:** chalcone synthase, *Chrysanthemum nankingense*, genome-wide analysis, pattern of expression

## Abstract

The *chalcone synthase* (*CHS*) gene family catalyzes the first committed step in the biosynthesis of flavonoids and plays key roles in various biological processes in plants. However, systematic studies of the *CHS* gene family in chrysanthemum remain unknown to date. In this study, 16 *CnCHS genes* were identified by searching the complete genome sequence of *Chrysanthemum nankingense*. Most contained two exons and one intron with Chal-sti-synt_N and Chal-sti-synt_C domains. A phylogenetic tree of *CnCHSs* indicated divergence into three major groups, including I, II, and III. Analyses of the genes and promoters of these genes indicated that there are many *cis*-acting elements that respond to light, phytohormones, stress, and developmental stages. The *CnCHS* genes have extensive patterns of expression in various tissues and stages of flower development. Tandemly repeated and segmental repeat genes were expressed at higher levels in different tissues than most of the *CnCHS* genes that have been identified. *CnCHS10* is expressed at higher levels in various flower organs than in vegetative tissues, particularly in disc floret petals and pistils. Our study provides valuable information for the systematic analysis of the *CnCHS* gene family, which also contributes to further research on flavonoid synthesis and petal colors of chrysanthemum.

## 1. Introduction

Flavonoids are important active components that contain a C6-C3-C6 carbon skeleton that is derived from phenylalanine, and they play essential roles in many biological processes and responses to biotic and abiotic stress in plants [1]. Flavonoids are also known as natural antioxidants. They have antibacterial, anti-inflammatory, analgesic, and antipyretic properties. Extensive studies on flavonoid biosynthesis have been conducted in several plants, including *Arabidopsis thaliana* [2], maize (*Zea mays*) [3], and petunia (*Petunia hybrida*) [4], and a number of structural and regulatory genes involved in this process have been identified. CHS is the first dedicated enzyme that catalyzes the initial committed step of the flavonoid biosynthetic pathway. CHS catalyzes the condensation of three malonyl-CoA molecules with one 4-coumaroyl-CoA to produce the precursor of different flavonoids, designated naringenin chalcone with a C13 carbon skeleton [5,6].

The CHS enzyme is known as a type III polyketide synthase (PKS) superfamily with stilbene synthases (STS) and 2-pyrone synthase (2PS), which has been found in all plants [7]. CHS consists of two conserved structural domains and has highly conserved amino acid sequences. The upper domain is located in the buried active site with four amino acids (Cys164, Phe215, His303, and Asn336), which are defined as the catalytic machinery, while the lower domain is adjacent to the upper domain and has a large active site that is required for chalcone formation [5,6]. Only a few amino acid modifiers can significantly alter the three-dimensional arrangement of the catalytic center that enables them to efficiently bind and modify the substrate, which can also result in decreasing activities or redirected functions of CHS [8]. CHS enzymes have also been demonstrated to be components of a multigene family with a different number of copies in various plants owing to gene duplication events and subsequent positive selection [9,10,11]. At least 3 gerbera CHS genes had been found in *Gerbera* hybrid [12]; 8 or more CHS genes have been found in petunia [13], and 14 CHS genes have been identified in the genome of maize [14]. Different copies of CHS have evolved to be functionally divergent in varying tissues or developmental stages [12]. Thus, a systematic study of the CHS gene family is crucially important to understand the synthesis of flavonoids and petal pigments in plants.

Chrysanthemums are economically important cut flowers and pot plants and are widely prized in the world flower market. The dry capitulum and leaves of chrysanthemums are also a form of traditional Chinese medicine and herbal tea, and their use has been well-documented in TCM to minimize heat and toxic materials. They are used to treat headaches, dizziness, the common cold, and dim eyesight. Modern pharmacological studies have proven that chrysanthemums have various pharmacological activities, such as antioxidative [15], anti-inflammatory [16], cardiovascular protective [17], antibacterial and antiviral [18], and immunoregulatory activity [19], because they contain high amounts of flavonoids, such as linarin, diosmetin-7-glucoside, and tilianin; phenolic acids, such as isochlorogenic acid C, isochlorogenic acid A, and 1,3-dicaffeoylquinic acid; and volatile oils, such as juniper camphor, borneol, and *cis*-caryophyllene [18,20,21,22]. Furthermore, flavonoids are not just the primary active components in chrysanthemums; as active substances, they also serve as the major pigments for flowers, as well as having a role in stress tolerance [23,24].

There is a remarkably significant positive correlation between the activity of CHS and contents of flavonoid pigments, as well as those of other flavonoids in plants [25]. The levels of expression of CHS became much higher in dark-purple ray florets compared with the light-purple ray florets at the later stages of capitulum development [23]. The expression of CHS first increased and then decreased with the increase in flowering degree in *Chrysanthemum morifolium* cv. ‘Hangju.’ However, there was no significant correlation among anthocyanins and the CHS gene [26]. CHS was expressed in both pink and white flowers, hence Wang et al. et al. [27] suggested that the lack of anthocyanin in white flowering cultivars cannot be due to the blockage of CHS expression. In contrast, *GCHS1* is the major CHS enzyme in gerbera petals, which is expressed more highly during pigmentation, and the silencing of *GhCHS1* expression resulted in petals that turned white; all the major flavonoid products decreased in the white petals [12]. A distinct phenotype has been attributed to different copies of CHS, but to our knowledge, no systematic studies on the CHS family in chrysanthemums have been conducted to date.

Recently, the whole-genome assembly of *C. nankingense* has been completed, which has provided more scope for the genome-wide identification of *CHS* gene family to analyze their evolutionary processes and functional diversification [28]. In this study, the *CnCHS* genes were identified from *C. nankingense*. We then investigated their sequence information, phylogenetic relationships, conserved motifs, and divergence information, and the patterns of expression of these *CnCHS* genes in different organs were also evaluated based on transcriptome analyses. The results of this study will provide the foundation for a comprehensive analysis of the *CHS* genes family in chrysanthemums.

## 2. Materials and Methods

### 2.1. Identification of CnCHS Family Members in the Chrysanthemum Genome

The complete gene and protein sequences of *C. nankingense* were downloaded from the Chrysanthemum genome database (http://www.amwayabrc.com/index.html (accessed on 10 August 2022)) [28]. The Hidden Markov Model (HMM) profiles of the Chal_sti_synt_C (PF00195) and Chal_sti_synt_N (PF02797) domains were downloaded from the Pfam protein family database (http://pfam.xfam.org/ (accessed on 15 August 2022)) [29]. The conserved domain sequences of the CHS family were obtained based on a BLAST program with a maximum threshold e-value of 10^−5^ using the CHS profiles as queries [30]. All the predicted sequences were aligned based on Cluster W [31], and these redundant sequences were discarded. The sequences were predicted using the Pfam database [29], NCBI Conserved Domain Search Service (http://forestry.fafu.edu.cn/db/PhePacBio/blast/blast_cs.php (accessed on 17 August 2022)) [32] and SMART (http://smart.embl-heidelberg.de/ (accessed on 17 August 2022)) [33] to confirm the presence of both the Chal_sti_synt_C and Chal_sti_synt_N domains in the predicted sequences, which was crucial for the identification of valid CHS proteins. In addition, we estimated the molecular weight (MW), isoelectric point (pI), instability index, and hydrophilicity of each protein using ExPASy (http://www.expasy.org/tools/ (accessed on 18 August 2022)) [34], while the subcellular localization information was predicted using the WoLF PSORT program (https://www.genscript.com/wolf-psort.html (accessed on 18 August 2022)) [35].

### 2.2. Analysis of Gene Structure, Conserved Motifs, and Sequence Alignment

The exon–intron structures of each *CnCHS* were illustrated by comparing their cDNAs with genome sequences using Gene Structure Display Server (GSDS 2.0) (http://gsds.gao-lab.org/ (accessed on 22 August 2022)) [36]. The conserved motifs in each putative CnCHS protein were identified using the MEME program (http://meme-suite.org (accessed on 25 August 2022)) [37] with the following parameters: the minimum width of motif was 6; the maximum width was 200; the maximum number of motifs was 10; and the e-value < 1 × 10^−20^.

Currently, the crystal structure of alfalfa (*Medicago sativa*) CHS (MsCHS) has revealed the architecture function of the active site on the cyclization reaction that leads to the synthesis of chalcone in plants. We used the secondary structure of MsCHS as a template to analyze the secondary structures of chrysanthemums CHSs. An alignment of MsCHS and all the CnCHS proteins was performed using the ClustalW 2.0.10 software. After that, highly conserved residues were marked using GeneDoc 2.7 software. Moreover, promoters were obtained from 2000 bp of the upstream sequences of the putative *CnCHS* genes based on the Chrysanthemum genome, which were used to predict the *cis*-regulatory elements within the promoter regions using Plant CARE (http://bioinformatics.psb.ugent.be/webtools/plantcare/html/ (accessed on 2 September 2022)) [38].

### 2.3. Phylogenetic Tree Construction of the CnCHS Genes

All the selected CHS protein sequences from chrysanthemums and *A. thaliana*, gerbera, common morning glory (*Ipomoea purpurea*), petunia, tomato (*Solanum lycopersicon*), maize, and tobacco (*Nicotiana tabacum*) were used for the phylogenetic analysis. The sequences were aligned using the ClustalX 1.83 software with default parameters [31]. The phylogenetic tree was then constructed with MEGA X [39] with a bootstrap of 1000 times to study the systemic evolution of CnCHS members in detail. These selected sequences were classified into different subfamilies based on the topological relations of the phylogenetic tree obtained. Subsequently, the online tool EvolView v3 (http://www.evolgenius. info/evolview/ (accessed on 7 September 2022)) [40] was used to edit the phylogenetic tree.

### 2.4. Scaffold Localization and the Calculation of Ka/Ks Values

The physical map was drawn to show the distribution of CnCHS genes on different scaffolds of chrysanthemum using MapChart 2.32 software [41] based on their positions on the chrysanthemum scaffold. Duplicate gene pairs were obtained by aligning the paralogs of chrysanthemum CHS using BLAST-v2.9.0+ program. Pairwise alignment of the identified duplication gene pairs of CDS sequences was performed using ClustalW 2.1. Potential gene duplications were analyzed based on the following major criteria: (a) the length of alignable sequence covered >75% of the longer genes; and (b) the similarity of aligned regions >75% [42]. Duplicated genes on a single scaffold with no more than one intervening were defined as tandem duplications. Otherwise, they were defined as segmental repeats in this study. The Ka_Ks calculator Toolbox 2.0 software [43] was used to calculate the synonymous substitution rate (Ks) and nonsynonymous substitution rate (Ka) values based on the coding sequence alignments to estimate the selective pressure that the protein has been subjected to. The genes were considered to have experienced positive directional selection with values >1 or purifying selection <1 and as neutral nonadditive gene with values equal to one. The time of divergence was further calculated using the formula T = Ks/2r [44], and r was considered to be 1.5 × 10^−8^ synonymous substitutions per site per year for the *CnCHSs* of dicotyledonous plants based on the fossil pollen data analyses [45].

### 2.5. Analysis of the Profile of Expression of Identified CnCHS Genes

Published raw RNA-seq data from different tissues or developmental phases of Chrysanthemum were downloaded from the NCBI SRA database (PRJNA548460) for the analysis of expression of the *CnCHS* genes [46]. In addition, the PRJNA548460 dataset contained all the vegetative tissues, various flower organs, and flower buds of different stages of the chrysanthemum cultivar ‘Jinba.’ The clean reads were used to map the *C. nankingense* reference genome using BWA-MEM2 [47] and Bowtie2-2.5.0 software [48]. The levels of gene expression were estimated using the fragments per kilobase of transcript per million mapped reads (FPKM) values with TopHat 2.0.14 and Cufflinks 2.2.1 based on the read count data of the gene expression in each sample obtained [49]. Each sample was tested with three replications, and an FPKM > 1.0 in at least one sample of putative *CnCHS* genes was utilized to calculate the mean average. The average value of the expression of each sample was showed in a heatmap using the Heatmapper program (http://www.heatmapper.ca (accessed on 27 September 2022)) [50].

## 3. Results

### 3.1. Identification of the CHS Genes in Chrysanthemums

Based on the *C. nankingense* reference genome, 16 nonredundant *CHS* genes were identified using the Chal_sti_synt_C and Chal_sti_synt_N domains by querying the *C. nankingense* genome database using the HMMER search. The details of gene name, chromosomal position, location coordinates, protein information, and cell location are described in Table S1. This demonstrated that all the *CnCHS* identified were mapped on 15 scaffolds, and *CnCHS6* and *CnCHS7* were located on the same scaffold (utg31268_pilon_pilon). In addition, the lengths of CHS proteins ranged from 197 to 401 amino acids. The molecular weights ranged from 21,773.13 to 43,864.65 Da, and the pI ranged from 5.17 to 9.46 in chrysanthemums. The prediction of protein subcellular location indicated that only CnCHS3 was localized in the nucleus. Five *CnCHS* genes were targeted to the chloroplast, and 10 *CnCHS* genes were targeted to the cytoplasm by calculating the increment of diversity and finite coefficient of diversity using amino acids.

### 3.2. Sequence Alignment of Alfalfa MsCHS2 and the CnCHS Identified

**Figure 1:**
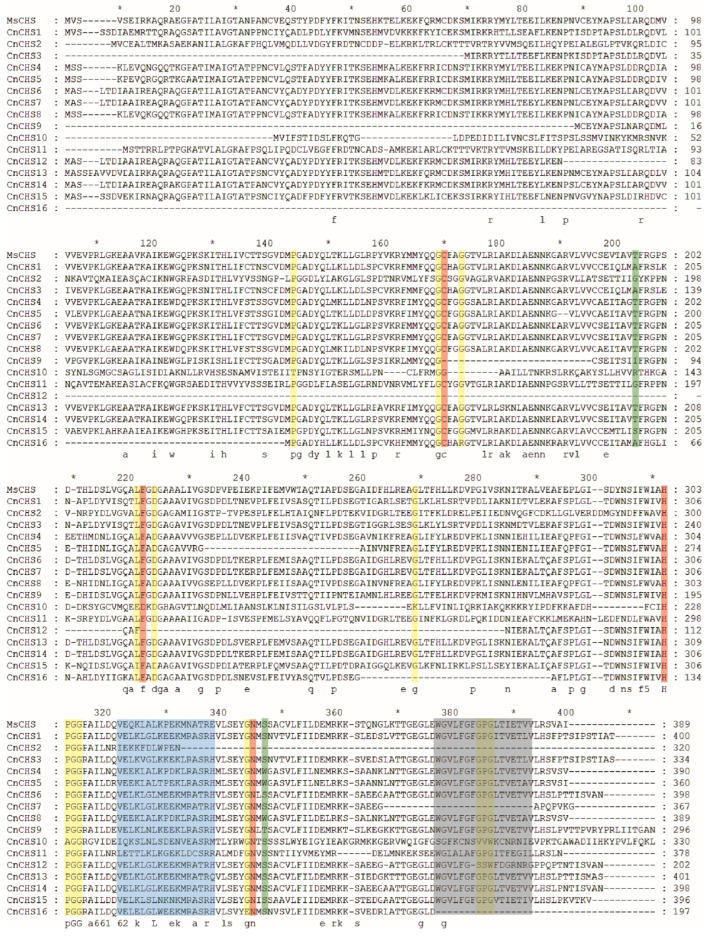
A comparison of amino acid sequences between alfalfa MsCHS2 and CnCHS. Functionally important conserved residues are marked with differently colored backgrounds. The four catalytic residues are highlighted with red. Inactive active catalytic sites are highlighted with yellow. Important residues that affect the binding pocket volume and the cyclization pocket are highlighted with green. Malonyl-CoA binding sites and signature sequences are highlighted with blue and gray, respectively. The amino acids are numbered based on those of alfalfa MsCHS2.

Alignment of the amino acids in the alfalfa MsCHS2 and CnCHS revealed that most of the CnCHS identified contained all previously reported active-site amino acids with four amino acids, including Cys164, Phe215, His303 and Asn336, and inactive active catalytic sites of the CHS enzymes (Figure 1). Several active amino acids, including Cys164, Phe215, or Asn303, were absent in CnCHS2, CnCHS9, and CnCHS12. In contrast, Cys164 and Phe215 were replaced with other amino acids in CnCHS10. In addition, most CnCHS sequences also contained malonyl-CoA binding sites and other signature sequences of CHS enzymes, which are required for the formation of chalcones. These results suggested that all the *CnCHS* genes identified have important conserved sequences of the CHS enzyme, while the amino acid substitutions in different *CnCHS* genes could lead to their functional diversity.

### 3.3. Phylogenetic Analysis and Classification of CnCHS

**Figure 2:**
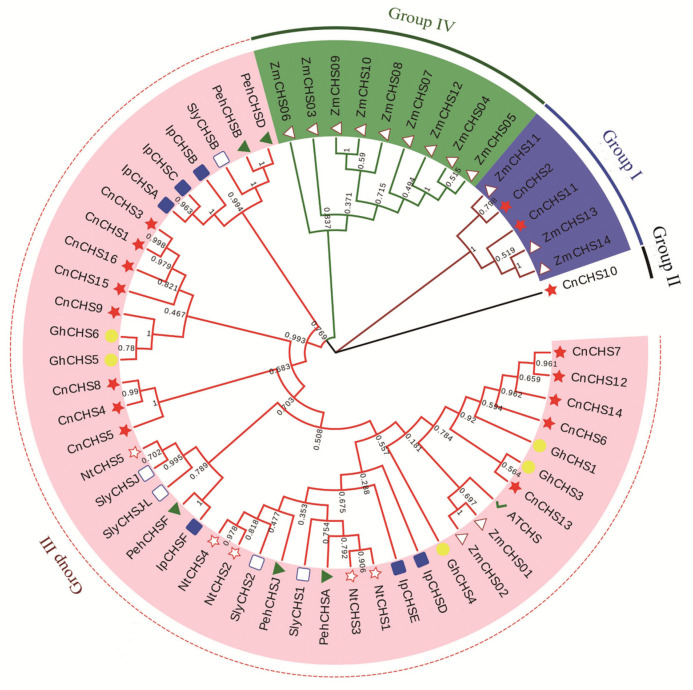
Phylogeny of selected *CHS* genes based on their neighbor-joining distance. *AtCHS* = *Arabidopsis* chalcone synthase, *GhCHS* = *G.* hybrid chalcone synthase, *PehCHS* = petunia chalcone synthase, *IpCHS* = common morning glory chalcone synthase, *SlyCHS* = tomato chalcone synthase, *NtCHS* = tobacco chalcone synthase, *ZmCHS =* maize chalcone synthase.

To investigate the phylogenetic relationships among the *CnCHS* genes identified, we used a neighbor-joining method to construct a phylogenetic tree with 57 amino acid sequences retrieved from different plants. Based on domains and the features, all these selected amino acid sequences could be classified into five major groups, namely Groups I–IV (Figure 2). All the *CHS* sequences retrieved from *A. thaliana*, gerbera, petunia, common morning glory, tomato, and tobacco, but not maize, were distributed in three groups. Group III contained the largest number of 13 *CnCHS* genes; Group I contained *CnCHS2 and CnCHS11*, and the remaining gene, *CnCHS10*, was distributed separately in Group II. A further phylogenetic analysis indicated that *CnCHS6*, *CnCHS7*, *CnCHS12*, *CnCHS13,* and *CnCHS14* were closely related to *GhCHS1* and *GhCHS3* that originated from the same branch, which demonstrated that *C. nankingense* is the closest relative to gerbera rather than several other plants.

### 3.4. Gene Structure and Motif Analysis of CnCHS

**Figure 3:**
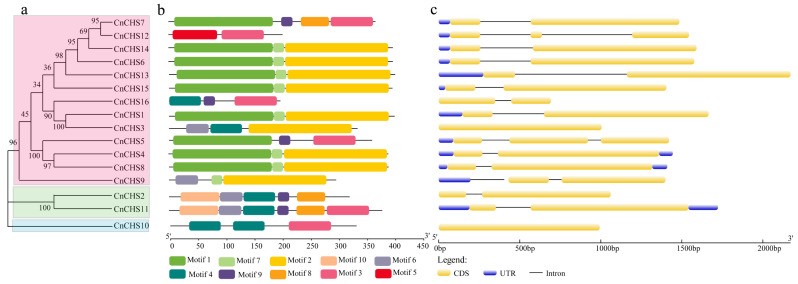
Phylogenetic analysis, gene structures, and motif analysis of the *CnCHS* genes. (**a**) Phylogenetic tree of the *CnCHS* genes. (**b**) Motif analysis of *CnCHS* members. Differently colored blocks represent different motifs. (**c**) Gene structure of the *CnCHS* genes. The blue blocks represent the upstream/downstream the yellow blocks represent the coding sequences (CDS), and the black lines represent the introns.

The conserved motifs of CnCHS proteins were analyzed using MEME program. Ten conserved motifs (Motif 1–10) were identified in the CnCHS proteins, and each CnCHS protein contained different numbers of motifs that ranged from 2 to 6. The conserved motifs 1, 4–7, and 10 were located in the Chal-sti-synt-N domain, while motifs 2, 3, 8, and 9 were located in the Chal-sti-synt-C domain (Figure 3b). Although none of the CnCHS contained all 10 motifs, all the CnCHS proteins contained the Chal-sti-synt-N and Chal-sti-synt-C domains. Interestingly, unique motif 10 was only found in *CnCHS2* and *CnCHS11* that are members of Group I, and the repeated motif was only identified in *CnCHS10*, which is a member of Group II (Figure 3a,b). To further investigate the structures, the intron/exon structures of the *CnCHS* genes were predicted by an alignment of cDNA to genomic sequences, and the results were visualized using the Gene Structure Display Server (GSDS). The number of exons ranged from 1 to 3 among these *CnCHS* genes, and the same subfamilies shared similar conserved motifs and intron/exon structures (Figure 3a,c). Most *CnCHS* genes had two exons, except for *CnCHS3* and *CnCHS10*, which only had one exon. *CnCHS5* and *CnCHS12* had three exons. This suggests that these three *CnCHS* genes could have experienced a unique evolutionary process that resulted in them having different functions.

### 3.5. Chromosomal Location and Gene Duplication

**Figure 4:**
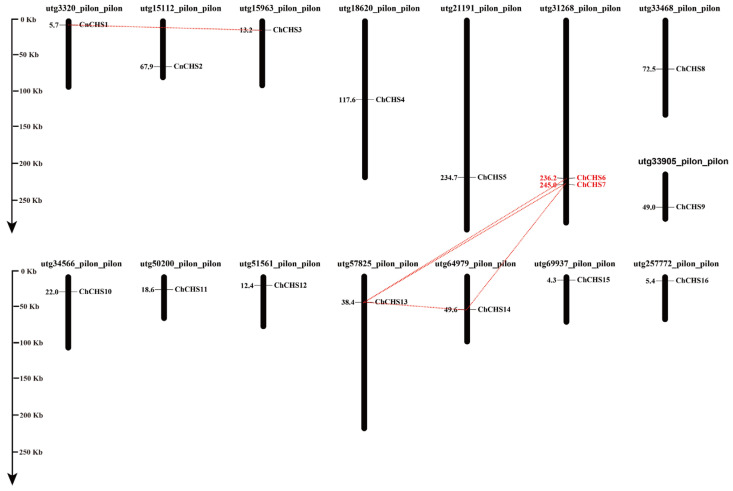
Location of 16 *CnCHS* genes on chrysanthemum scaffolds. The segmental duplicated genes are connected by red dashed lines, and the tandemly repeated genes are marked in red.

To determine the genomic distribution of the *CnCHS* genes, these *CnCHS* genes that were identified were mapped on their corresponding scaffold by searching the released database of *C. nankingense*. The results showed that all the *CnCHS* genes identified mapped to 15 *C. nankingense* scaffolds (Figure 4). Segmental duplication analyses of the *CnCHS* genes indicated that two *CnCHS* genes were identified as tandemly repeated, which was attributed to the location of *CnCHS6* and *CnCHS7* on the same scaffold utg_31268_pilon_pilon genes with highly homologous amino acid sequences. In addition, five gene pairs (*CnCHS1/CnCHS3*, *CnCHS6/CnCHS13*, *CnCHS7/CnCHS13*, *CnCHS7/CnCHS14* and *CnCHS13/CnCHS14*) were found to be involved in segmental repeats. They are generally more closely related to each other, with a high similarity, and shared a similar evolutionary process (Figure 2).

The duplication events over the past several million years are considered as a driving force for the expansion of many gene families during the evolutionary process. To understand the selective constraints among the *CnCHS* genes, the *Ka* and *Ks* values of five segmental duplications and one tandem duplication were calculated using the KaKs_calculator. In our study, the *Ka*/*Ks* ratios of six *CnCHS* gene pairs <1, indicating that these genes had undergone purifying selection at a low evolutionary rate rather than positive selection, and this selection would eliminate deleterious mutations in chrysanthemums (Table 1). Moreover, the Ks values were used to calculate the time of divergence of eight gene pairs, which ranged from 6.06 to 206.14 million years ago (Mya).

### 3.6. Promoter Region Analysis of CnCHS Genes

**Figure 5:**
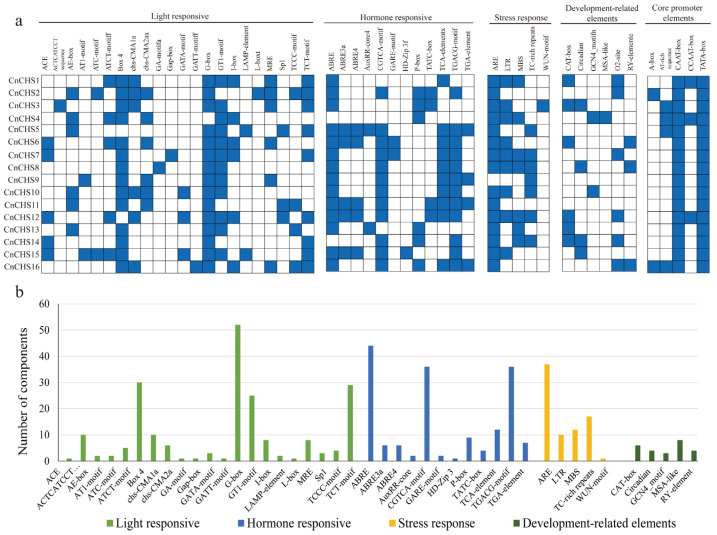
*Cis*-element analysis in the promoter of *CnCHS* genes. (**a**) The black blocks at the top show five different types of *cis*-acting elements, and the horizontal axis represents the different *cis*-acting elements. (**b**) Numbers of four different types of *cis*-acting elements.

Multiple *cis*-acting elements in the promoters of *CnCHS* genes are described (Figure 5). In addition to the core promoter elements, the well-characterized TATA and CAAT boxes, four categories of *cis*-regulatory elements, including light responses, hormone responsive, stress response, and developmental-related elements, were found to be highly represented in the promoter region of *CnCHS* genes. A total of 22 types of elements of light-related responses were abundant in the *CnCHS* genes (Figure 5a,b). Each *CnCHS* promoter had 4–10 light-responsiveness elements, while *CnCHS2* and *CnCHS15* contained more light-responsiveness elements (Figure 5a), which suggested that they differentially responded under light compared with the other *CnCHS* genes. The G-box was commonly found in the CnCHS genes in greater numbers than any other elements (Figure 5b), which is a general regulatory element of the responses of plants to environmental stimuli. Mutation of the G-box had been reported to result in *COX5B-2* without the function of responding to UV-B induction in *A. thaliana* [47]. A total of 12 types of hormone response elements were involved in various hormones, such as ABRE, ABRE3a and ABRE4, which were found to be involved in the response to abscisic acid (ABA). AuxRR-core and TGA-elements were involved in the response to auxin (AUX). CGTCA and TGACG motifs were involved in the response to methyl jasmonate (MeJA). The GARE-motif, P-box, and TATC-box were involved in the response to gibberellin (GA), and the TCA-element was involved in the response to salicylic acid (SA) (Figure 5a,b). Most *CnCHS* genes contained ABRE, the CGTCA motif, TCA element, and TGACG motif, which suggested that AUX, MeJA, and SA facilitate the expression of CnCHS genes. In addition, ARE responded to anaerobic induction, and the LTR element responded to low temperature and was ubiquitous in most of the *CnCHS* genes promoters. The fourth class of development-related elements consisted of the CAT-box, Circadian, GCN4_motif, MAS-like, O2-site, and RY-element, which were involved in meristem expression, cell cycle regulation, zein metabolism regulation, seed-specific regulation, and cell cycle regulation. In conclusion, these *CnCHS* genes could be related to light, hormones, stress responses, and growth pathways, which is inconsistent with the functions of flavonoids.

### 3.7. Analysis of the Expression of CnCHS Genes

**Figure 6:**
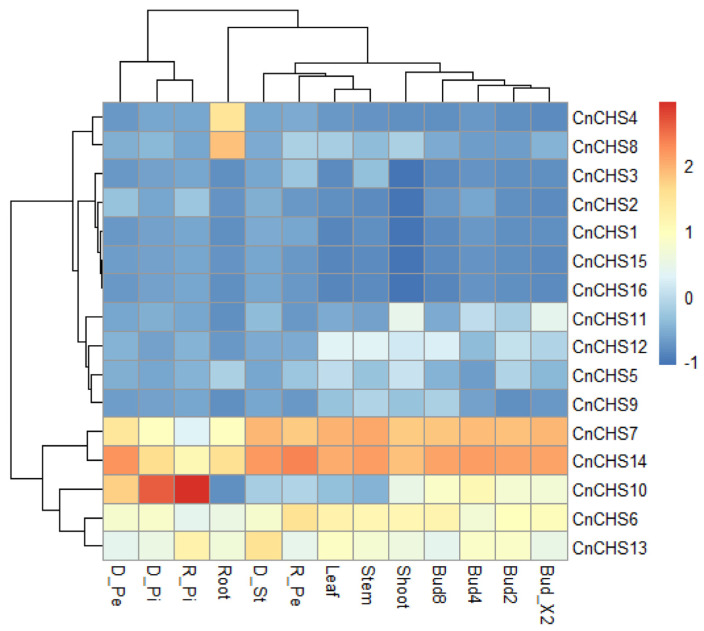
Profiles of expression of the *CnCHS* genes in all vegetative tissues, various flower organs, and flower buds of different stages of chrysanthemum ‘Jinba.’ There were three replicates per sample Log2-transformed average FPKM values, as per the standardization, were used to plot the heatmap. Red indicates high expression, and green indicates low expression. Bud2, 4, 8, and Bud_X2: flower buds of different stages. D_St: disc floret stamen; D_Pe: disc floret petal; D_Pi: disc floret pistil; R_Pe: ray floret petal; R_Pi: ray floret pistil.

To explore the patterns of gene expression of the *CnCHS* genes in different tissues, we comprehensively performed the previously reported transcriptome profiling of all the vegetative tissues, various flower organs, and flower buds of different stages of chrysanthemum ‘Jinba.’ Among these *CnCHS* genes, most were minimally expressed in diverse organs of chrysanthemum ‘Jinba’ (Figure 6). It is notable that *CnCHS1*, *CnCHS2,* and *CHS15* were expressed at low levels with FPKM values < 1. *CnCHS16* was usually silent and was not expressed in the vegetative tissue and floral organs of chrysanthemum ‘Jinba’. The FPKM values are shown in Supplementary Table S2. In contrast, several other *CnCHS* genes, including *CnCHS6*, *CnCHS7*, *CnCHS13,* and *CnCHS14*, were expressed at higher levels in each sample (Figure 6). These four *CnCHS* genes were identified as tandemly repeated genes or segmental repeats (Figure 4), which had a close evolutionary relationship with the *GhCHS1* of gerbera (Figure 2). Moreover, *CnCHS4* and *CnCHS8* were more highly expressed in the roots than in other tissues. Interestingly, *CnCHS10* was expressed at higher levels in various flower organs and flower buds of different stages than in vegetative tissues, particularly in the disc floret petals and ray floret pistils. The pattern of expression suggested that the *CnCHS* genes play diverse biological functions in different tissues and developmental phases.

## 4. Discussion

A total of 4950 types of CHS belong to a supergene family, and the copy number of the CHS gene varies substantially among different plants, resulting in diverse functions [51]. CHS is pivotal for the biosynthesis of flavonoid antimicrobial phytoalexins and anthocyanin pigments. Given the versatility of the CHS genes, a comprehensive identification and functional validation of the CHS family are expected to enrich databases in the field of chrysanthemum flower development and pigment metabolism. In this study, 16 chrysanthemum CHS genes were identified with the Chal_sti_synt_C (PF00195) and Chal_sti_synt_N (PF02797) domains (Figure 1), and their sequences were highly homologous to the well-characterized CHS from alfalfa. Although the CHS enzyme is encoded as a single copy in *A. thaliana* and nearly all taxa [20], it is considered that gene duplications and segmental repeats have led to the wide expansion of the gene family during the evolutionary process [52,53]. In general, the CHS enzyme is typically encoded by a gene family in plants. For example, a family of 3 *GCHS* genes has been found in gerbera, 6 *CHS* genes were identified in common morning glory [51], and at least 8 or 10 complete *CHS* genes were cloned and sequenced from petunia [13]. The genome of *C. nankingense* has gone through multiple evolutionary mechanisms, such as whole-genome duplication (WGD) or polyploidization and locally repetitive genome expansion [28]. As a result, 1 tandemly repeated gene and 5 gene pairs of segmental duplication events were found among the 16 *CnCHS* genes (Figure 4). That suggested that segmental duplication could be the dominating contributor for a gene family of 16 *CHS* genes in *C. nankingense*.

All the *CnCHS* genes identified could be broadly classified into three major classes based on sequence alignment and an analysis of phylogenetic relationships (Figure 1 and Figure 2). Furthermore, the motif distribution and gene structure of the *CnCHS* genes were similar in the same subgroup, which resulted in a conserved evolution shared in some *CnCHS* genes (Figure 3). Moreover, the conserved motifs 1, 4–7, and 10 represented the Chal-sti-synt-N domain, while motifs 2, 3, 8, and 9 comprised the Chal-sti-synt-C domain. The catalytic triad of CHN is considered to be very important for the catalytic function and was distributed in each *CnCHS* gene identified. That indicated that the catalytic triad inherited from the KAS III ancestor is highly conserved in all the *CnCHS* genes. All the *CnCHS* genes contain the CHS family-specific Pro375 (Figure 3), which also indicates their conserved evolution, although there are several substitutions at positions Phe215 and Phe265, which probably resulted in a different choice of the substrate. These substrates are connected with CoA binding. Importantly, the sequence alignment of these identified *CnCHS* genes with those of *MsCHS* were highly similar, which suggests that the CHS family is conserved in different species. In addition, the analysis of gene structure further proved the phylogenetic analysis. It also supported the hypothesis that the *CnCHS* genes were highly conserved during the evolutionary process. In addition, most *CnCHS* genes contain two exons and one intron, which is consistent with the previously proposed structure that is composed of two exons and one intron in maize [14]. It also proved the conservation of *CHS* among different plants.

In our study, *CnCHS6* and *CnCHS7* were identified as tandemly repeated, as well as five gene pairs that were found to be involved in segmental repeats (Figure 4). A previous study suggested that a whole-genome triplication (WGT1) occurred ~57 Mya, and the most recent WGD occurred ~5.8 Mya in the genome of *C. nankingense*, except for the ancestral palaeohexaploidy (WGT-γ) event that occurred 122–164 Mya in all eudicots [27]. In general, tandem duplications contribute to generate new genes and diverse functions, such as the WGD event, which led to an enhancement in photosynthetic capacity, improved the productivity with respect to biomass, and resulted in differentially transcribed genes in chrysanthemums based on a comparison between the diploid and tetraploid forms of *C. nankingense* [15]. However, segmental duplications tend to disperse gene copies that result in the slow evolution of genes. In this study, the presence of segmental duplications among these *CnCHS* genes indicates that the chrysanthemum *CHS* gene family is conserved and slowly evolving.

The patterns of gene expression are an important reflection of gene function, and genes with common features that possess a common origin are likely to possess a similar pattern of expression [54,55]. Along with multiple functions of *CHS* genes, the expression of these genes can change under specific environments or is specific to an organ or developmental stage. In this study, the transcription profiles of chrysanthemums revealed that the levels of gene expression were highly diverse among tissues and in different developmental periods. Most of the *CnCHS* gene family members were poorly expressed in most tissues; in contrast, several *CnCHS* genes, designated *CnCHS6*, *CnCHS7*, *CnCHS13,* and *CnCHS14,* had a higher level of expression in various tissues (Figure 6). Several *CnCHS* genes exhibited variable patterns of expression that suggest that functional diversification took place among these *CnCHS* genes. The result of separately knocking down *GhCHS1* and *GhCHS4* in gerbera inflorescences by virus-induced gene silencing (VIGS) showed that only *GhCHS1* was contributing to flavonoid biosynthesis [12]. A phylogenetic analysis showed that these four *CnCHS* genes had a close evolutionary relationship with the *GhCHS1* of gerbera (Figure 2). Moreover, flavonoids were isolated and identified in the roots, stems, leaves, and flowers of chrysanthemum [56]. It has been deduced that *CnCHS6*, *CnCHS7*, *CnCHS13,* and *CnCHS14* are involved in flavonoid biosynthesis in chrysanthemum but were irrelevant to the synthesis of anthocyanidins. Our analysis of the pattern of expression indicated that *CnCHS4* and *CnCHS8* had a higher level of expression in roots that could play a role in biological processes related to the roots. In addition, *CnCH10* is expressed at higher levels in various flower organs, particularly in disc floret petals and pistils than in vegetative tissue. Due to CHS genes expressed in both ray florets of a pink flowering (cv. H5) and two white flowering (cvs. Keikai and Jinba) Chrysanthemum grandiflorum cultivars, Chen et al. [57] suggested that the higher expression of CHS did not contribute to anthocyanin accumulation. Jo et al. [23] also suggested that the transcriptional regulation of the flavonoid biosynthetic pathway did not cause flavonoid accumulation. However, GCHS1 is an essential gene for pigmentation formed in gerbera petals, and silencing of GCHS1 expression resulted in petals turning to white [12]. In Asiatic hybrid lily (*Lilium* spp.) cultivar Lollypop, which develops bicolor tepals with pigmented tips and white bases, the expression of CHS genes expressed higher in pigmented tepal parts than those in nonpigmented parts [58]. The expression of *CHS* genes was similar in two Asiatic hybrid lily cultivars, ‘Montreux’ (pink tepals with spots) and ‘Connecticut King’ (yellow tepals without spots). The expression of three CHS genes was not detected in unpigmented leaves, stems, and white bulb scales, but it was detected in bulb scales in which anthocyanin pigmentation had been induced [59]. Moreover, in flower organs, three CHS genes were expressed in the anthocyanin-pigmented tepals, filaments, and pistils of ‘Montreux.’ In unpigmented filaments and pistils of ‘Connecticut King,’ the pigmented anthers only accumulated *LhCHSC* mRNA in both cultivars. It was concluded that the expression of *CnCH10* is involved in the distinct pigmentation pattern in flower organs. Moreover, many genes exhibited similar patterns of expression, which could in turn catalyze similar substrates in the same biochemical pathway. Although our expression-profiling analyses provide important information to study the function of the CnCHS gene family in flavonoid biosynthesis and the anthocyanidin synthesis of chrysanthemum, further experiments are required to confirm the function of these *CnCHS* genes that have been identified.

## 5. Conclusions

CHS is a key enzyme in the synthetic process of flavonoids. We identified and analyzed the features of expression of 16 *CnCHS* genes (*CnCHS1*–*16*) from *C. nankingense* to explore their potential functions. The result of gene structures, evolutionary patterns, and the profiles of expression of these *CnCHS* genes identified suggest that the *CnCHS* gene is conserved and slowly evolving, but the specific genes function differently during evolution. According to our systematic analysis, *CnCHS6*, *CnCHS7*, *CnCHS13,* and *CnCHS14* are probably involved in flavonoid biosynthesis in chrysanthemum rather than anthocyanidin synthesis. In contrast, *CnCH10* is likely to be involved in a distinct pattern of pigmentation. Our study extends the knowledge of *CnCHS* genes and will contribute to research about the molecular evolution, expression, and regulation of these genes. More functional analysis will need to be conducted to further characterize the exact functions of these *CnCHS* genes identified in *C. nankingense*.

## Figures and Tables

**Table 1:** Calculation of Ka, Ks, and Ka/Ks and the time of divergence of CnCHS gene pairs.

Duplicated Gene Pairs	Ka	Ks	Ka/Ks	Duplicated Type	Time (Mya)
*CnCHS1*\*CnCHS3*	0.03	0.10	0.29	Segmental	7.82
*CnCHS6*\*CnCHS7*	0.01	0.10	0.09	Tandem	7.68
*CnCHS6*\*CnCHS13*	0.05	2.39	0.02	Segmental	179.24
*CnCHS7*\*CnCHS14*	0.01	0.08	0.12	Segmental	6.06
*CnCHS7*\*CnCHS13*	0.06	2.73	0.02	Segmental	204.72
*CnCHS13*\*CnCHS14*	0.06	2.75	0.02	Segmental	206.14

## Data Availability

The genome data were downloaded from the Chrysanthemum genome database (http://www.amwayabrc.com/index.html (accessed on 13 September 2022)), the transcriptome data were downloaded from the NCBI SRA database with Bioproject numbers PRJNA548460 (https://ncbi.nlm.nih.gov/bioproject/?term=PRJNA548460 (accessed on 13 September 2022)).

## Supplementary Materials

The following supporting information can be downloaded at: https://www.mdpi.com/article/10.3390/genes13112145/s1, Supplementary Table S1. The chalcone synthase family genes of *C. nankingense,* Supplementary Table S2. The FPKM values of identified *CnCHS* genes.
