# Supplementary material for: Genome-Wide Identification, Characterization, and Expression Analysis of CHS Gene Family Members in Chrysanthemum nankingense
Source: Genes (Basel). 2022 Nov 18;13(11):2145. doi: 10.3390/genes13112145 (PMC9690667; doi:10.3390/genes13112145)
Supplement: Supplementary file 1 [file genes-13-02145-s001.zip › Supplementary Table S1.pdf]

**Supplementary Table S1**. The chalcone synthase family genes of *C. nankingense*

| Gene name | Gene ID | Chromosomal position | Location coordinates | Predicted Protein information | | | Predicted subcellular location |
|---|---|---|---|---|---|---|---|
| | | | | Mw (Da) | Size (aa) | pI | |
| CnCHS1 | CHR00061989-RA | utg3320_pilon_pilon | 5699 - 7363 | 43792.77 | 400 | 5.82 | cytoplasm |
| CnCHS2 | CHR00068181-RA | utg15112_pilon_pilon | 67925 - 68985 | 35183.43 | 320 | 5.17 | cytoplasm |
| CnCHS3 | CHR00062939-RA | utg15963_pilon_pilon | 13243 - 14247 | 36615.35 | 334 | 5.29 | Nucleus |
| CnCHS4 | CHR00027878-RA | utg18620_pilon_pilon | 117603 - 119046 | 42931.55 | 390 | 6.14 | chloroplast |
| CnCHS5 | CHR00018287-RA | utg21191_pilon_pilon | 234722 -236142 | 39667.79 | 360 | 8.17 | cytoplasm |
| CnCHS6 | CHR00019175-RA | utg31268_pilon_pilon | 236204 - 237780 | 43550.25 | 398 | 5.79 | chloroplast |
| CnCHS7 | CHR00019176-RA | utg31268_pilon_pilon | 244981 - 246465 | 40432.75 | 367 | 6.47 | chloroplast |
| CnCHS8 | CHR00047213-RA | utg33468_pilon_pilon | 72474 - 73883 | 42800.38 | 389 | 5.87 | cytoplasm |
| CnCHS9 | CHR00077726-RA | utg33905_pilon_pilon | 49032 - 50430 | 32458.79 | 296 | 6.06 | cytoplasm |
| CnCHS10 | CHR00059250-RA | utg34566_pilon_pilon | 21965 - 22957 | 36976.17 | 330 | 9.46 | chloroplast |
| CnCHS11 | CHR00080912-RA | utg50200_pilon_pilon | 18557 - 20279 | 41666.91 | 378 | 5.85 | chloroplast |
| CnCHS12 | CHR00073521-RA | utg51561_pilon_pilon | 12425 - 13968 | 22686.91 | 202 | 6.44 | cytoplasm |
| CnCHS13 | CHR00028844-RA | utg57825_pilon_pilon | 38412 - 40582 | 43864.65 | 401 | 6.11 | cytoplasm |
| CnCHS14 | CHR00062418-RA | utg64979_pilon_pilon | 49591 - 51181 | 43551.28 | 398 | 6.04 | cytoplasm |
| CnCHS15 | CHR00077616-RA | utg69937_pilon_pilon | 4299 - 5702 | 43438.20 | 396 | 7.16 | cytoplasm |
| CnCHS16 | CHR00079924-RA | utg257772_pilon_pilon | 5445 - 6136 | 21773.13 | 197 | 5.18 | cytoplasm |
